# Ventilator-Associated Pneumonia: An Update on the Role of Lung Ultrasound in Adult, Pediatric, and Neonatal ICU Practice

**DOI:** 10.1177/29768675251349632

**Published:** 2025-06-12

**Authors:** Diana Adrião, Francesco Mojoli, Rebeca Gregorio Hernandez, Daniele De Luca, Belaid Bouhemad, Silvia Mongodi

**Affiliations:** Department of clinical-surgical, diagnostic and pediatric sciences, 19001 Università di Pavia, Pavia, Italy; Department of Intensive Care, 679493 Unidade Local de Saúde Gaia e Espinho, Vila Nova de Gaia, Portugal; Department of clinical-surgical, diagnostic and pediatric sciences, 19001 Università di Pavia, Pavia, Italy; Anesthesia and Intensive Care, Fondazione IRCCS Policlinico San Matteo, Pavia, Italy; Neonatology Division, 16483 Gregorio Marañón University Hospital, Madrid, Spain; Division of paediatrics and Neonatal Critical Care, 27048 APHP-Paris Saclay University, Paris, France; Physiopathology and Therapeutic Innovation Unit-INSERM U999, Paris Saclay University, Paris, France; Department of Anaesthesiology and Intensive Care, C.H.U. Dijon, Dijon Cedex, France; 439716 Department of Anesthesiology and Intensive Care, Université Bourgogne Franche-Comté, Dijon, France; Anesthesia and Intensive Care, Fondazione IRCCS Policlinico San Matteo, Pavia, Italy

**Keywords:** ventilator-associated pneumonia, lung ultrasound, lung ultrasound aeration score, lung monitoring, lung aeration, pneumonia, quantitative lung ultrasound

## Abstract

Ventilator-associated pneumonia (VAP) remains one of the most common and challenging intensive care unit (ICU)-acquired infections, significantly contributing to mortality, morbidity, and healthcare costs. The diagnosis relies on quantitative analysis of a deep microbiological sample; a combination of clinical and radiological signs is commonly used to raise VAP suspicion in clinical practice. Traditional imaging methods such as chest radiography and computed tomography have limitations in critically ill patients under mechanical ventilation. Lung ultrasound (LUS) has emerged in the last years as a valuable tool in the assessment and monitoring of critically ill patients, including for diagnosis and management of VAP, due to its noninvasive bedside applicability and absence of radiation exposure. This last quality is of particular interest in the specific population of children and newborns, where radiation exposure should be further avoided. LUS allows for daily monitoring of lung aeration and provides a quantitative assessment through the LUS aeration score; an unexpected increase of LUS aeration score may raise the suspicion of superinfection. Key ultrasonographic findings, such as subpleural consolidations and consolidations with dynamic linear–arborescent air bronchogram, improve diagnostic specificity for VAP. Similarly to what happens with traditional radiology, the Ventilator-associated Pneumonia Lung Ultrasound Score (VPLUS) combines ultrasound signs with clinical parameters like purulent secretions to enhance diagnostic accuracy. Furthermore, LUS aeration score plays a crucial role in monitoring the response to treatment, enabling assessment of lung reaeration over time. It helps differentiate between treatment responders and nonresponders, guiding therapy adjustments and identifying complications.

This review highlights the evolving role of LUS in the early diagnosis, monitoring, and treatment of VAP across various ICU settings, including its application in adult, pediatric, and neonatal care.

## Introduction

Ventilator-associated pneumonia (VAP), one of the most frequent intensive care unit (ICU)-acquired infections, is defined as an infection of the pulmonary parenchyma occurring in patients who have undergone invasive mechanical ventilation for more than 48 h.

The epidemiology and diagnostic criteria for VAP are still controversial. The incidence of VAP is reported to range from 5% to 40%, with significant variations influenced by geography, type of ICU, and diagnostic criteria applied.^1^

The overall attributable mortality of VAP is around 13%, with higher rates observed in surgical ICU patients and those presenting with mid-range severity scores at admission, such as an APACHE II score of 20 to 29 or a SAPS II score of 35 to 58.^2^ VAP also results in significant morbidity, as evidenced by prolonged duration of mechanical ventilation, and extended ICU and hospital stay.^3^ Beyond its clinical impact, VAP imposes a substantial economic burden, with healthcare costs rising by an estimated $40,000 per patient with VAP.^4^

Accurate diagnosis and timely treatment play a crucial role in improving VAP outcomes. While chest x-rays and computed tomography (CT) are commonly used for conventional VAP evaluation, these imaging techniques are often impractical for real-time monitoring in critically ill patients. Consequently, lung ultrasound (LUS) is increasingly being adopted as an effective tool for assessing VAP in the ICU setting.

This review aims to explore the role of LUS in the diagnosis and management of VAP, emphasizing its potential advantages, current evidence, and practical applications in the ICU setting.

## Diagnosis of VAP

VAP develops due to impaired airway defenses and alterations in microbial flora. Endotracheal intubation disrupts protective mechanisms such as coughing and mucociliary clearance, allowing microaspiration of pathogen-containing secretions,^5–8^ that spread bilaterally with positive inspiratory pressure, finally reaching the alveoli.^5^

The diagnosis of VAP remains a challenging and controversial topic, with no established consensus on the most accurate diagnostic criteria. Several factors contribute to these difficulties, including the wide range of conditions in ICU patients that can cause fever or elevated white blood cell counts, challenges in distinguishing between bacterial airway colonization and actual infection, and the inherent limitations of portable chest imaging in the ICU setting.^9^

No single clinical criterion, biomarker, or scoring system has proven sufficiently accurate for diagnosing VAP. Consequently, VAP should be suspected whenever new signs of respiratory deterioration potentially attributable to infection arise. Traditionally, the diagnosis of VAP is based on the simultaneous presence of 3 key criteria^1,3,10^:

1. Clinical suspicion, indicated by signs such as the onset of fever/hypothermia, purulent endotracheal secretions, leukocytosis or leukopenia, increased minute ventilation, worsening oxygenation, or heightened vasopressor requirements.
2. Radiographic evidence, typically new or progressive and persistent infiltrates on chest imaging.
3. Microbiological confirmation, through positive cultures obtained from lower respiratory tract specimens, such as endotracheal aspirates or bronchoalveolar lavage.

### Clinical Diagnosis of VAP

Preliminary signs of inflammation, such as fever, tachycardia, and leukocytosis, while raising suspicion of VAP, are nonspecific and can be seen in any process associated with the systemic inflammatory response of critical illness (Table 1).^5^ Although clinical examination remains essential, it has limited diagnostic accuracy for VAP, with fever showing in a recent systematic review a sensitivity of 66.4% (95% CI [40.7-85.0]) and specificity of 53.9% (95% CI [34.5-72.2]), and purulent secretions demonstrating a sensitivity of 77.0% (95% CI [64.7-85.9]) and specificity of 39.0% (95% CI [25.8-54.0]).^11^

**Table 1.** Clinical, radiological, and microbiological criteria for diagnosing VAP.

Criteria	Description
Clinical	Fever > 38 °C or hypothermia
	Leukocytosis or leukopenia
	New onset or change in sputum
	Cough, dyspnea, or tachypnea
	Worsening oxygenation
Radiological	New or progressive infiltrates on chest radiography or computed tomography
	Consolidation with dynamic linear-arborescent air bronchogram or subpleural consolidations on LUS
Microbiological	Positive quantitative or semiquantitative culture from lower respiratory tract specimen

Abbreviations: VAP: ventilator-associated pneumonia; LUS: lung ultrasound.

In critically ill patients, cough suppression due to sedation, positive pressure ventilation, and artificial airways, combined with impaired mucociliary clearance and bacterial colonization of airway devices, can lead to a high burden of purulent tracheal secretions.^5,6^ These secretions may indicate lower respiratory tract infection but can also arise from tracheobronchitis, smoking history, or underlying respiratory conditions. Alterations in oxygenation, ventilation, or gas exchange may signal lung inflammation suggestive of VAP but can also reflect noninfective causes. VAP and acute respiratory distress syndrome (ARDS) frequently overlap diagnostically, as pneumonia is a leading cause of ARDS, while ARDS itself increases the risk of developing VAP.^12^

Scores combining clinical and radiological parameters have been proposed to enhance diagnostic accuracy, with the Clinical Pulmonary Infection Score (CPIS; Table 2)^13^ being the most commonly used; however, recent guidelines do not recommend CPIS for diagnosing VAP.^10^

**Table 2.** The clinical pulmonary infection score (CPIS)*.

Temperature (°C)	
≥ 36.5 and ≤ 38.4	0
≥ 38.5 and ≤ 38.9	1
≥ 39.0 or ≤ 36.0	2
Blood leukocytes per mm^3^	
≥ 4000 and ≤11,000	0
< 4000 or >11,000	1
Either < 4000 or > 11,000 plus band forms ≥ 500	2
Tracheal secretions	
Absence of tracheal secretions	0
Presence of nonpurulent tracheal secretions	1
Presence of purulent tracheal secretions	2
Oxygenation: PaO_2_/FiO_2_, mm Hg	
> 240 or ARDS	0
≤ 240 and no evidence of ARDS	2
Chest radiography	
No infiltrate	0
Diffuse (or patchy) infiltrate	1
Localized infiltrate	2
Culture of tracheal aspirate	
Pathogenic bacteria cultured in rare or light quantity or no growth	0
Pathogenic bacteria cultured in moderate or heavy quantity	1
Pathogenic bacteria cultured in moderate or heavy quantity plus same pathogenic bacteria seen on Gram stain	2

Abbreviations: VAP: ventilator-associated pneumonia; ARDS: acute respiratory distress syndrome.

* CPIS varies from 0 to 12 points. A score > 6 indicates a high likelihood of VAP.

## Radiological Diagnosis of VAP

VAP has historically been evaluated using chest radiography as the primary imaging modality due to its portability, enabling bedside assessment, and relatively low cost. Nevertheless, chest radiography has significant limitations for diagnosing pneumonia in patients receiving mechanical ventilation in the ICU, with a recent systematic review reporting a sensitivity of 88.9% (95% CI [73.9-95.8]) and a specificity of 26.1% (95% CI [15.1-41.4]).^11^

The interpretation of chest radiography in critically ill patients is often challenging due to the overlap of many common pathologies that present with lung infiltrates,^5^ frequently present at ICU admission^14^ and to technical limitations.^15^ Finally, repeated chest radiography can lead to excessive radiation exposure for ICU patients.

CT remains the gold standard for thoracic imaging.^16^ However, in mechanically ventilated patients, no specific CT sign of pneumonia has been described,^17^ and its primary utility lies in its negative predictive value.^18^ Additionally, its routine use is limited by several factors, including high radiation exposure, the risks associated with transporting critically ill patients to the radiology department, and elevated costs.

Given these challenges, there is an increasing need for a diagnostic method that is nonirradiating, noninvasive, easily repeatable, and suitable for bedside use in the ICU. LUS has emerged as a promising alternative, with growing evidence supporting its utility in both detecting and monitoring VAP.^18,19^

## Overview of LUS

Over the past decades, LUS has progressively emerged as a leading imaging modality in emergency departments and intensive care units. Its rapid availability, bedside applicability, and radiation-free nature have made it an invaluable tool for the reliable assessment of critically ill patients.^20,21^ The unique features of LUS make it ideal for repeated examinations, thus providing a powerful tool for ongoing patient monitoring.^22,23^

When combined with clinical signs and symptoms, LUS has demonstrated superior diagnostic performance compared to chest radiography in mechanically ventilated patients, including those with VAP.^24^ Studies indicate that incorporating LUS into routine ICU practice led to a 26% reduction in chest radiography use and a 47% reduction in CT scans, thereby significantly reducing radiation exposure.^25,26^

Notably, LUS has also been adopted in neonatal and pediatric critical care settings, as endorsed by recent guidelines from an international consensus conference, highlighting its broad applicability across different age groups and critical care environments.^27^

LUS can be used both for the early diagnosis of VAP and for its monitoring.

## LUS in VAP Diagnosis

In the emergency department, LUS is as a reliable tool for the early diagnosis of community-acquired pneumonia in adults,^28^ where the visualization of a consolidated lobe is highly specific for the diagnosis. LUS detection of consolidations in this setting yields a sensitivity of 93.4% and a specificity of 97.7%.^29^ However, this is not always true for mechanically ventilated patients suspected of having VAP.

In adult critically ill, mechanically ventilated patients, pulmonary infiltrates are common and may result from various causes, including noninfectious etiologies such as derecruitment and atelectasis.^18^ Consequently, in these patients, the presence of a tissue-like pattern alone is insufficient for a definitive diagnosis of VAP, as severe loss of aeration can result from multiple mechanisms, particularly in dependent lung regions. Therefore, while lung consolidations detected by LUS in these patients have high sensitivity for VAP, their specificity remains low.^19,30,31^ In fact, a prospective multicenter study demonstrated that the presence of a tissue-like pattern alone had a sensitivity of 93% but a specificity of 0%.^19^ In this context, interpreting additional signs, such as the presence of an air bronchogram, can improve diagnostic accuracy.

**Figure 1.**
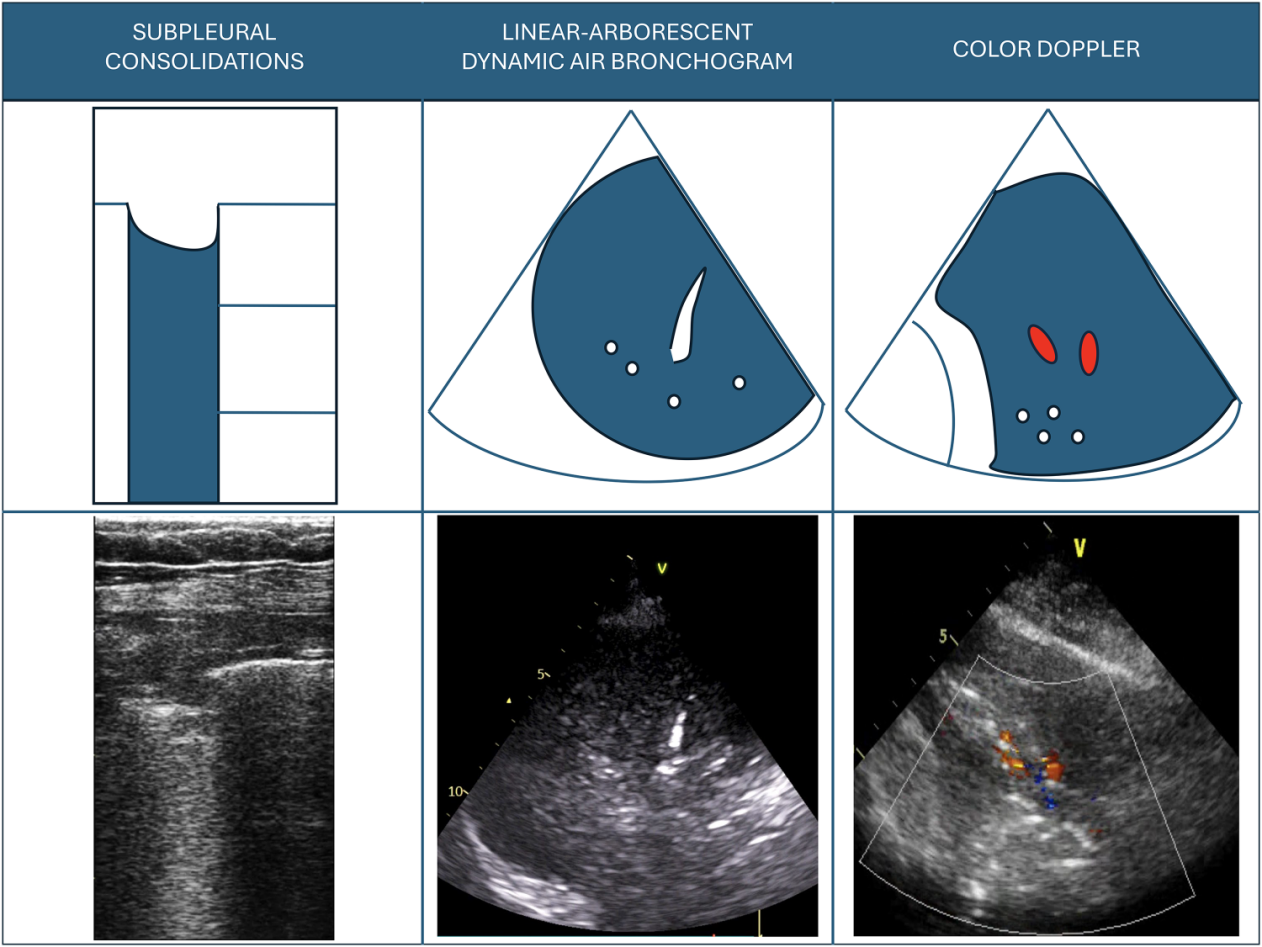
Ultrasound signs visualized in ventilator-associated pneumonia.

A dynamic air bronchogram corresponds to air moving synchronously with tidal ventilation in the distal airways, effectively ruling out obstructive atelectasis.^32^ The shape of the dynamic air bronchogram can be either punctiform or linear-arborescent. A punctiform dynamic air bronchogram excludes atelectasis but lacks specificity for VAP,^33^ as it may also be associated with other conditions, such as derecruitment with patent airways. If the air bronchogram is dynamic and linear-arborescent, it has been shown to be highly specific for VAP diagnosis, with the visualization of a single consolidated region containing a linear-arborescent dynamic air bronchogram yielding a specificity of 81% (Electronic Supplementary Material ESM 1; Figure 1).^19,34^

A second ultrasound sign associated with VAP is the presence of subpleural consolidations, visualized as echo-poor regions adjacent to the pleura with a diameter of at least 0.5 cm (ESM 2; Figure 1). They demonstrated good sensitivity (81%) but low specificity (41%) for VAP, as they can also be present in lung contusions, ARDS, and pulmonary embolism.^19^

These findings were integrated into a simple score known as the Ventilator-associated Pneumonia Lung Ultrasound Score (VPLUS; Table 3), which combines ultrasonographic signs with a clinical parameter (purulent secretions). VPLUS outperformed the classical CPIS, even when CPIS was combined with a direct examination of tracheal aspirate.^19^ The simultaneous presence of both subpleural consolidations and dynamic linear-arborescent air bronchogram demonstrated a high specificity (88%) and a high positive predictive value (86%) for VAP diagnosis. Notably, when all VPLUS criteria (VPLUS 4) were met, the specificity reached 100%, with a positive predictive value of 100%, making it a reliable bedside tool for identifying high-risk VAP cases and guiding early antibiotic therapy.^19^

**Table 3.** Ventilator-associated pneumonia lung ultrasound score.

Parameter	Points
Purulent tracheal secretions	1
≥ 2 Areas with subpleural consolidations	1
≥ 1 Area with consolidation and a dynamic linear-arborescent air bronchogram	2

In ARDS patients, while subpleural consolidations alone lack sensitivity for diagnosing VAP—since they are part of the typical ARDS pattern—the appearance of a dynamic linear-arborescent air bronchogram within a consolidation is highly specific for VAP.^35^ Additionally, LUS has proven valuable for bedside identification of pulmonary superinfections in COVID-19 patients admitted to the ICU. A retrospective observational study showed that dynamic linear-arborescent air bronchogram within lobar or hemilobar consolidations have very high specificity for detecting pulmonary overinfection in these patients, mostly if newly appeared in a patient daily monitored with LUS.^34^

Although the dynamic linear-arborescent air bronchogram is a highly specific sign, false negatives can occur. When this sign is absent, no definitive conclusion about the cause of the consolidation can be drawn (ESM 3). In such cases, a second LUS examination following disobstructive fiberbronchoscopy has been suggested to detect previously obscured air bronchogram.^21,36^ This approach may help improve the early diagnosis of VAP when an initial nonspecific pattern, such as a static or absent air bronchogram, is observed. However, further validation in larger populations is still required.

Color Doppler ultrasound can be used for identifying blood vessels within lung consolidations, with pneumonia often displaying a characteristic tree-like vascular pattern^37-39.^ During lung infections, the inflammatory process suppresses hypoxic pulmonary vasoconstriction, allowing the intrapulmonary shunt (perfusion of nonaerated lung parenchyma) to become visible.^38,40,41^ While lung vascular flow assessment is not quantitative, the identification of a major vessel within a consolidation strongly suggests a significant shunt, indicating a substantial impact of the consolidation on oxygenation.^42^ In contrast, atelectasis is typically characterized by reduced regional pulmonary blood flow due to hypoxic pulmonary vasoconstriction, making the intrapulmonary shunt either absent or challenging to detect.^43^

Recent studies have utilized an extended LUS approach that incorporates the assessment of color Doppler flow and dynamic air bronchogram, demonstrating high overall diagnostic accuracy for detecting pneumonia in different ICU populations (ESM4).^41,44^ While the dynamic linear-arborescent air bronchogram has been shown to be highly specific for VAP, the presence of pulsatile flow on color Doppler imaging has demonstrated high sensitivity.^37,44^

## Quantitative LUS and VAP

While a qualitative approach provides important information on the morphological assessment of the lung for the diagnosis of respiratory conditions, a quantitative approach enhances the utility of LUS by enabling the quantification of lung loss of aeration and the daily monitoring of lung status.^45^ This quantitative approach relies on the computation of the LUS aeration score, which allows clinicians to evaluate the severity of lung disease at the time of hospital admission and monitor changes in lung aeration on a daily basis.

It distinguishes 4 progressive steps of loss of aeration according to the artifacts visualized^46–51^:

- Score 0: Normal aeration (A-lines or no more than 2 B-lines).
- Score 1: Mild loss of aeration (3 or more well-spaced B-lines, coalescent B-lines, and/or subpleural consolidations occupying ≤ 50% of the pleural line).
- Score 2: Moderate loss of aeration (well-spaced B-lines, coalescent B-lines, and/or subpleural consolidations occupying > 50% of the pleural line).
- Score 3: Severe loss of aeration (tissue-like pattern with a thickness > 2.5 cm).

This comprehensive examination is performed in 12 standard thoracic regions, 6 on each side of the thorax (anterior, lateral, and posterior fields are identified by sternum, anterior, and posterior axillary lines; each field is divided into superior and inferior regions).^23^ The total score is obtained by summing the regional scores and ranges from 0 (all regions are well-aerated) to 36 (all regions are consolidated).^21,45,47,48^

Quantitative LUS enables monitoring of disease progression and evaluation of treatment efficacy in mechanically ventilated patients. Moreover, daily LUS assessment allows clinicians to track changes in lung aeration over time, facilitating the early detection of VAP signs.^33,52^

While the LUS score is not specific for VAP, it reliably reflects overall lung aeration. A significant increase in the LUS score has been shown to correlate with worsening lung aeration and should raise suspicion of VAP, especially when accompanied by clinical signs such as purulent secretions.^26,53^ Notably, studies in COVID-19 patients with VAP have demonstrated that LUS scores increased significantly compared to baseline measurements taken 48 to 72 h prior to the diagnosis.^34,53^

LUS can also be used to monitor VAP recovery and assess antibiotic efficacy. In a previous study, a reaeration score was computed after one week of antibiotic therapy to quantify changes in lung aeration, allowing differentiation between responders and nonresponders to treatment.^54^ The global reaeration score is obtained by summing each regional reaeration score. Effective antibiotic therapy is associated with a positive global reaeration score, reflecting improved lung aeration, while a negative score indicates persistent or worsening loss of aeration. Specifically, a global reaeration score of +5 or higher corresponds to a lung volume gain of at least 400 mL, signifying treatment success, whereas a score of −10 or lower correlates with a lung volume loss of at least 400 mL, indicating treatment failure.^54^

This quantitative approach to monitoring VAP recovery offers several advantages. It allows early detection of antibiotic efficacy, aids in determining the appropriate duration of antibiotic therapy, and helps identify complications such as abscesses or septated effusions.^18^ Moreover, traditional chest radiography has been shown to be poorly accurate in detecting changes in lung aeration following antimicrobial therapy.^54^ In contrast, LUS-guided monitoring of lung reaeration correlates strongly with findings from quantitative CT scans, making LUS a reliable and practical bedside alternative to more complex imaging modalities.^18,54^ Indeed, a recent study evaluated the role of LUS and procalcitonin levels in guiding the discontinuation of antibiotic therapy in patients with VAP. The LUS reaeration score demonstrated a highly significant negative correlation with procalcitonin levels on Day 7 (*r* = −0.718, *P* < .001) and a highly significant positive correlation between reaeration observed by LUS and CT (*r* = 0.747, *P* < .001).^55^

## LUS in Pediatrics and Neonates

VAP rates in the pediatric population range from 2% to 32% according to the literature,^56^ making it the second most prevalent invasive infectious disease after bloodstream infections in most pediatric and neonatal ICUs (PICUs/NICUs).^57^ The 2018 CDC criteria are the most widely used globally.^58^ These criteria include a worsening of lung imaging with new infiltrates or opacifications after at least 48 h of invasive mechanical ventilation that were not previously present. This has been obviously suggested to be done with chest x-rays, but LUS can provide the same information with higher accuracy and suitability.

In complex and chronically ill neonates, such as those with bronchopulmonary dysplasia or congenital heart defects, differentiating the causes of respiratory worsening, including VAP, can be particularly challenging. These patients can have loss of lung aeration for underlying reasons and not only because of VAP: the distinction is even more difficult than in adult ARDS patients. In fact, the dynamic linear-arborescent air bronchogram is not always detectable in neonates and small infants, and the appearance of consolidation of infectious or noninfectious origin is relatively similar.^59^ The introduction of new ultra-high frequency small probes could theoretically improve the visibility of this sign and increase diagnostic accuracy.^60^ Laboratory tests have low specificity, and chest x-rays have poor reliability and accuracy.^61,62^ The use of CT scans is unfeasible for the high radiation exposure, the risk linked to the transfer of instable neonates and patient hypothermia. According to CDC criteria, in these patients, serial chest imaging (typically within a 7-day timeframe) must demonstrate persistent compatible findings to differentiate infectious from noninfectious pulmonary processes.^58^ This makes it especially important to find a noninvasive tool to evaluate lung parenchyma.

All this evidence, together with the favorable anatomical characteristics of pediatric patients, makes LUS a fundamental tool in modern PICUs/NICUs for the differential diagnosis of respiratory conditions.

The extensive development of LUS usage over the last 10 years in pediatric and neonatal critical care patients has led to international recommendations for using this point-of-care ultrasound tool. These guidelines suggest LUS as a valuable tool for diagnosing pneumonia in the pediatric population; however, they do not recommend specific LUS scoring systems or qualitative signs.^27^

Most of the available data on LUS and pediatric pneumonia focus on community-acquired pneumonia. Evidence suggests that LUS outperforms chest x-rays in diagnosing community-acquired pneumonia in the emergency department, helping to determine viral or bacterial etiology with high reliability while reducing exposure to ionizing radiation^63-66.^ LUS has also been shown to be as effective as CT for diagnosing necrotizing pneumonia and its complications, such as pleural effusion or necrosis, in pediatric patients.^67^ However, the evidence supporting LUS for diagnosing VAP remains limited.

Neonatal pneumonia typically presents with consolidations; however, no specific ultrasound features have yet proven useful in distinguishing among the various types—congenital, ventilator-associated, biliary, or community-acquired. Further dedicated research is needed in this field.

Some scores similar to the adult CPIS have been employed as the gold standard in some studies^68^ and others merging ultrasound and biological data,^57,68,69^ but there is no universally accepted scoring system.^70^ Similar to data in adults, these scores combining LUS findings, clinical parameters, and microbiological data generally show excellent diagnostic accuracy, with AUC values ranging from 0.86^68^ to 0.97^69^ in children and neonates. Nevertheless, most published studies are small and of relatively low quality.

Regarding LUS findings, these can be evaluated individually or as part of some LUS aeration score. Individually, the presence of B-lines demonstrates high sensitivity but very low specificity in the pediatric population, as B-lines are among the most common LUS findings. The combination of consolidations with clinical criteria shows both high sensitivity and specificity for diagnosing VAP.^57^ Unilateral lobar consolidations with dynamic air bronchogram represent the most specific finding for VAP across all studies (ESM 5). Other signs, such as pleural effusions or subpleural consolidations, are frequently described and may enhance the CDC scoring system by allowing earlier VAP diagnosis.^57^ This evidence suggests that LUS could replace chest x-rays for pediatric patients with suspected VAP.^57,71^ However, further studies comparing the 2 techniques head-to-head are needed.

To our knowledge, no studies have evaluated whether diagnosing VAP based on LUS findings can monitor antibiotic response or improve respiratory outcomes in critically ill pediatric patients. Recently, Guitart et al reported a 77% reduction in the number of chest x-rays per patient without significantly increasing costs in a randomized controlled trial. In this study, patients with suspected pneumonia were managed based on chest x-rays or LUS findings combined with procalcitonin levels.^71^ No studies have been published on the use of LUS aeration score for diagnosing or monitoring pediatric VAP.

## Limitations and Challenges

The effectiveness of LUS relies on the transmission of ultrasound beams through the chest wall to the lung surface.^21^ This transmission can be hindered by conditions such as subcutaneous emphysema, chest drains, or large thoracic dressings. Visualization also becomes particularly challenging in obese patients, where increased subcutaneous tissue may reduce image clarity. Moreover, as a bedside instrument in direct contact with patients, the ultrasound probe poses a risk of cross-contamination. Critically ill patients often harbor multidrug-resistant pathogens, and inadequate disinfection of the probe may facilitate the transmission of nosocomial flora.^72^ Although ultrasound machines are classified as noncritical items, their routine use in the ICU requires strict and standardized decontamination protocols to minimize infection transmission^72-74.^ Future research is needed to introduce noncontact ultrasound probes into clinical practice.

Another significant limitation is the incomplete visualization of the pleural surface. Studies indicate that LUS can directly assess approximately 70% of the pleural surface, with the remainder being obscured by the thoracic cage.^75^ Consequently, purely central pulmonary processes cannot be reliably detected or excluded using this technique. However, this rarely concerns critically ill patients.

Additionally, like other ultrasound techniques, LUS is operator dependent.^21,45^ Nonetheless, studies have shown excellent interoperator agreement in assessing lung aeration when performed by experienced operators.^46,47^ Therefore, adequate training and hands-on experience are essential to developing the advanced skills required for accurate interpretation and reliable integration of LUS findings into clinical management. No data are currently available on the level of training required to correctly identify different types of air bronchogram.^76^

## Conclusion

LUS is a reliable and valuable tool for the bedside diagnosis of VAP in critically ill patients. However, in ICU patients with preexisting lung injuries, the presence of lung consolidation alone is insufficient to confirm VAP, as similar patterns may be observed in other conditions such as atelectasis, derecruitment, or contusion.

The dynamic linear-arborescent air bronchogram is currently the only ultrasound sign with high specificity for VAP in patients of all ages. In experienced hands, the absence of pathological LUS findings can reliably rule out VAP, while identifying specific signs such as lobar or subpleural consolidations with a dynamic air bronchogram can confirm the diagnosis and support timely initiation of appropriate antibiotic therapy. Furthermore, LUS appears to be an accurate diagnostic tool for monitoring the respiratory effects of antimicrobial therapy in patients with VAP, allowing early detection of antibiotic-induced lung reaeration or progression of infection in cases of treatment failure.

Ultimately, the integration of specific ultrasonographic findings with clinical parameters improves diagnostic accuracy, reduces diagnostic uncertainty, and may potentially shorten the time to appropriate treatment. Further research should focus on refining LUS-based diagnostic algorithms and validating its use across different patient populations.

## Supplemental Material

**Figure:** Video 1. Linear–arborescent dynamic air bronchogram in an adult patient with ventilator-associated pneumonia

**Figure:** Video 2. Subpleural consolidation in an adult patient: echo-poor regions applied to the pleura

**Figure:** Video 3. Lobar consolidation with no air bronchogram; not specific for ventilator-associated pneumonia. Disobstructive fiberbronchoscopy is recommended

**Figure:** Video 4. Lobar consolidation with both arborescent air bronchogram and perfusion with color Doppler

**Figure:** Video 5. Linear–arborescent dynamic air bronchogram in a neonate with ventilator-associated pneumonia
